# Photocatalytic Activity of Y-Doped ZrO_2_ Thin Films

**DOI:** 10.3390/ijms27167487

**Published:** 2026-08-21

**Authors:** Carmen Mita, Mariana Frenti, Nicoleta Cornei, Georgiana Bulai, Daniela Pricop, Vasile Tiron, Marius Dobromir, Aleksandr S. Doroshkevich, Diana Mardare

**Affiliations:** 1 Faculty of Chemistry, “Alexandru Ioan Cuza” University of Iasi, 11 Carol I Blvd., 700506 Iasi, Romania; cmita@uaic.ro; 2 Faculty of Physics, “Alexandru Ioan Cuza” University of Iasi, 11 Carol I Blvd., 700506 Iasi, Romania; marianafrenti2009@gmail.com (M.F.); daniela.a.pricop@gmail.com (D.P.); 3 Integrated Centre for Environmental Science, Studies in the North-East Development Region–CERNESIM, “Alexandru Ioan Cuza” University of Iasi, 11 Carol I Blvd., 700506 Iasi, Romania; georgiana.bulai@uaic.ro; 4 Institute of Interdisciplinary Research, Department of Exact and Natural Sciences, Research Center on Advanced Materials and Technologies, “Alexandru Ioan Cuza” University of Iasi, 700506 Iasi, Romania; vasile.tiron@uaic.ro (V.T.); marius.dobromir@uaic.ro (M.D.); 5 Joint Institute for Nuclear Research, Dubna 141980, Russia; doroh@jinr.ru

**Keywords:** Y-doped ZrO_2_, ITO substrate, hydrophilicity, photocatalysis, Rhodamine B, methylene blue, oxygen vacancy

## Abstract

In this paper, we have investigated the photocatalytic performances of some yttrium-doped ZrO_2_ films for decomposing Rhodamine B and Methylene Blue. The as-deposited thin films (obtained by RF magnetron sputtering) were heat-treated to stabilize their structure. To assess the films’ characteristics, a group of analytical methods (XRD, XPS, AFM and DRS) allowed for the determination of their structural, surface, and optical properties. These characteristics were correlated with the observed photocatalytic activity and wetting behaviour. The Y-doped ZrO_2_ film with medium nanoparticle size and a high contribution of the oxygen vacancy is found to be more efficient in Rhodamine B and Methylene Blue photodegradation; the 100% degradation efficiency was reached in 70 min and 40 min, respectively, for the 3 mg/L solution dye. The photodegradation mechanism is driven by photogenerated holes, and a possible reaction mechanism was proposed. By investigating the charge carrier separation at the film–ITO interfaces, made through a comparative analysis of their determined band edge potentials, we conclude that the transfer is not possible in either of the semiconductor pairs, so ITO does not “help” the photocatalytic process.

## 1. Introduction

Persistent organic pollutants from the industrial sector (especially the textile industry), such as synthetic dyes, significantly degrade water quality and pose risks to human health [1,2,3,4,5]. Given their potential to form toxic compounds and generate eutrophication, the systematic management and removal of these dyes from industrial waste are vital for environmental protection [5,6,7]. The toxicity of synthetic dyes poses a severe risk to biological systems, as exemplified by Methylene Blue (MB) and Rhodamine B (RhB). Ingestion of MB can cause gastrointestinal distress, while acute exposure may lead to methemoglobinemia, severely impairing oxygen transport in the blood [4]. Similarly, RhB, a versatile dye used extensively in textiles, medicine, and as a food additive, exhibits dose-dependent toxicity [5,8,9]. Given that these organic pollutants are especially resistant to conventional chemical and biological treatments, there is an urgent need for “green” remediation strategies. In this context, photocatalytic oxidation appears to be a necessary solution for the effective degradation of these persistent contaminants in industrial wastewater.

Transition metal oxides (like TiO_2_, ZnO, WO_3_, ZrO_2_) have held their place as premier photocatalysts due to their unique electronic configurations, specifically their semiconducting properties and bandgap tunability. In these materials, the most prevalent defects are oxygen vacancies, which introduce energy levels in the forbidden band and serve as active reaction sites, fundamentally changing the photocatalytic properties of the material [10,11].

Among transition metal oxides, ZrO_2_ is distinguished by its excellent photocatalytic performance, chemical inertness, thermal stability, mechanical strength and high redox abilities [12]. To prevent structural failure caused by volume expansion during phase transitions (monoclinic to tetragonal/cubic), a method used to stabilize ZrO_2_ is by doping with yttrium. This stabilization preserves the tetragonal and/or cubic structures at low temperatures, removing the risk of cracking and structural collapse [13]. The larger ionic radius of Y^3+^ (0.90 Å for CN = 6 and 1.019 Å for CN = 8) compared with Zr^4+^ (0.72 Å for CN = 6 and 0.84 Å for CN = 8), according to Shannon’s effective ionic radii [14], produces local lattice distortions while preserving the structural compatibility required for substitutional incorporation. Moreover, the lower valence state of Y^3+^ requires charge compensation through the formation of oxygen vacancies, according to the Kröger–Vink relation:

(1) $$Y_{2}O_{3}\to {2Y}_{Zr}^{\prime }+V_{O}^{••}+3O_{O}^{x}$$

where $Y_{Zr}^{\prime }$ denotes a Y^3+^ ion occupying a Zr^4+^ site and carrying one effective negative charge, $V_{O}^{••}$ represents an oxygen vacancy with two effective positive charges, and $O_{O}^{x}$ denotes an O^2−^ ion occupying a regular oxygen site with zero effective charge.

The combined effect of lattice distortion and oxygen-vacancy formation decreases the free-energy difference between the monoclinic and tetragonal polymorphs, allowing the high-temperature tetragonal phase to be retained at room temperature [15,16].

Besides ensuring structural stabilization, the oxygen vacancies generated during yttrium incorporation also play an important role in the photocatalytic performance of ZrO_2_. They create surface defect sites, introduce localized electronic states within the bandgap, and can act as charge-carrier trapping centres, thereby suppressing electron–hole recombination and promoting photocatalytic reactions. Consequently, optimizing the yttrium concentration is expected to provide a balance between structural stability and photocatalytic efficiency [15,17,18].

The concentration of yttrium is an important parameter governing both the phase composition and defect chemistry of yttria-stabilized ZrO_2_. Previous studies have shown that increasing Y incorporation progressively suppresses the monoclinic phase and promotes the stabilization of the tetragonal and, at higher dopant concentrations, cubic or cubic-like structures [12,13,19]. This phase stabilization is directly related to the substitution of Zr^4+^ by Y^3+^, which requires charge compensation through the formation of oxygen vacancies. However, the effect of Y concentration is not limited to the amount of oxygen vacancies generated. Experimental and theoretical studies have demonstrated that the concentration of yttrium also affects the spatial distribution and local configuration of oxygen vacancies. For example, oxygen vacancies in yttria-stabilized zirconia (YSZ) have been reported to form preferential configurations with respect to the Y^3+^ ions, while vacancy–vacancy interactions and vacancy ordering become increasingly important with increasing Y concentration [15,20]. Studies of Y-doped ZrO_2_ thin films have also shown that increasing Y incorporation can influence both phase stabilization and oxygen-vacancy-related defects [15,17,18]. These findings indicate that Y concentration determines not only the phase assemblage of ZrO_2_ but also its local defect structure, with both factors potentially affecting its surface and functional properties.

Although the wide bandgap of ZrO_2_ usually restricts the material to being UV light-absorbent, its strong oxidation capacity and structural versatility ensure its role as a leading material for sustainable “green” treatment technologies.

Despite the extensive use of yttria-stabilized zirconia (YSZ) in structural, thermal, electrochemical, sensing and antibacterial applications [20,21,22], relatively few studies have investigated its photocatalytic behaviour [20,23,24,25]. The available reports are mainly focused on Y-doped ZrO_2_ nanopowders, where yttrium incorporation has been shown to enhance photocatalytic performance through tetragonal phase stabilization, oxygen-vacancy generation, and improved charge-carrier separation [21,24,25]. To the best of our knowledge, studies on the photocatalytic performance of Y-doped ZrO_2_ thin films are still limited, although immobilized photocatalysts offer important practical advantages, including easy recovery, high mechanical stability, and excellent reusability. Therefore, further investigations are needed to clarify the influence of yttrium incorporation on the photocatalytic performance of ZrO_2_ thin films.

To address this research gap, the photocatalytic properties of undoped and yttrium-doped ZrO_2_ thin films deposited by radio frequency magnetron sputtering (RFms) were investigated in the present work.

The main objectives of this study were to improve the photocatalytic properties of ZrO_2_ thin films and establish a direct correlation between yttrium-induced structural stabilization, oxygen vacancies, electronic band structure, and their photocatalytic activity. As a novel contribution, this study demonstrates that an optimum yttrium concentration and RFms method maximize charge-carrier separation and significantly enhance the photocatalytic degradation of Rhodamine B and Methylene Blue, providing new insights into the structure–property–performance relationships governing YSZ photocatalysts.

## 2. Results and Discussion

### 2.1. X-Ray Diffraction Analysis

**Figure 1:**
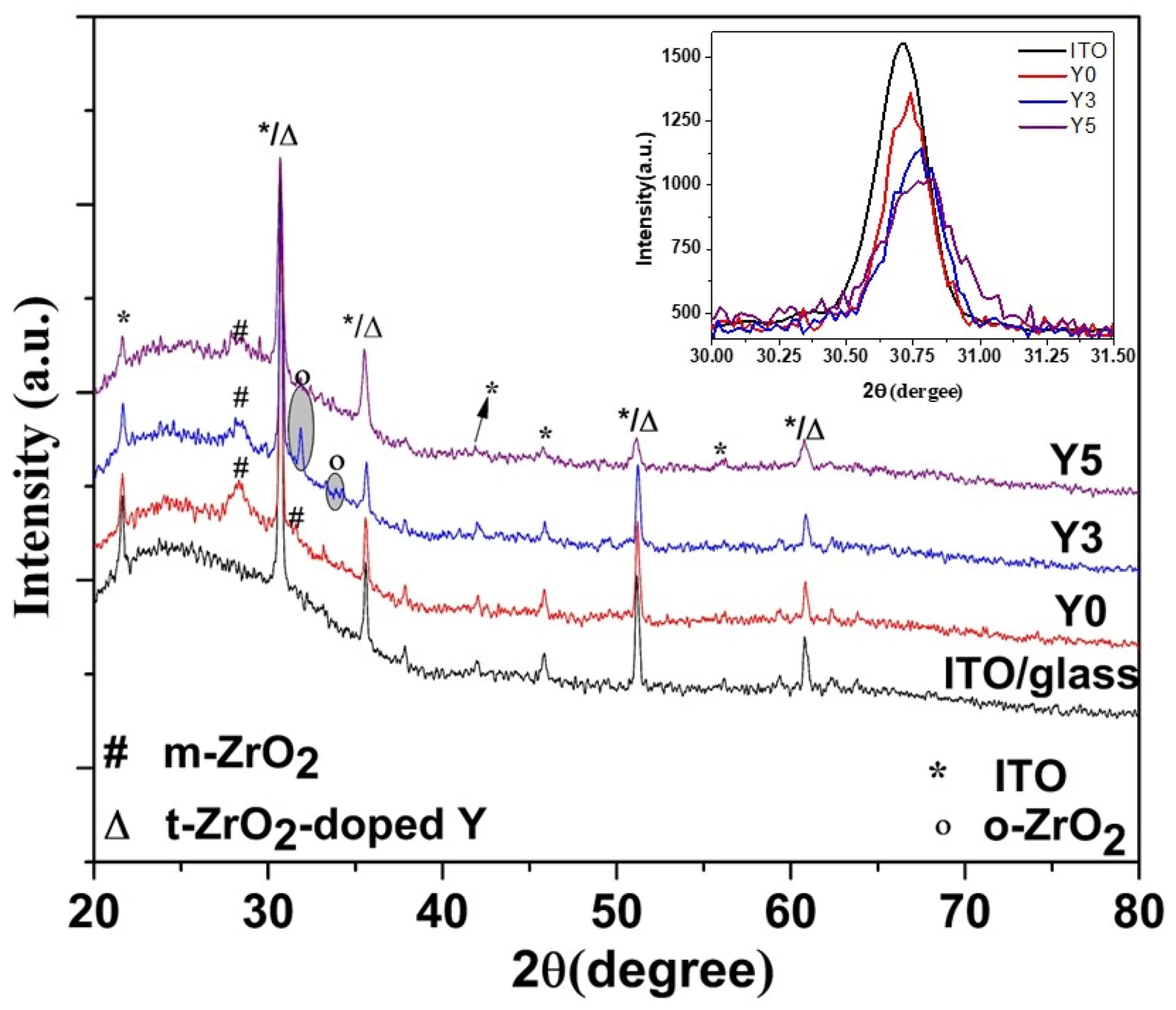
XRD patterns of the investigated films and of the ITO substrate.

In Figure 1 are given the X-Ray Diffraction (XRD) patterns of the investigated films and of the ITO substrate. Both the undoped film (Y0) and the Y-doped films (Y3, Y5) exhibit diffraction peaks characteristic of the monoclinic structure (m-ZrO_2_), corresponding to the *P12_1_*/*c1* space group (JCPDS card no. 98-008-2212). With the increase in Y content, the intensity of the monoclinic reflections gradually decreases, while their broadening becomes more pronounced, indicating a reduction in crystallite size and an increase in structural disorder induced by Y substitution. This is reflected in the average crystallite sizes of the doped samples, which decrease from 6.55 nm for the undoped film to 6.01 nm for the highest yttrium concentration (Table 1). Such refinement of the crystallites is commonly associated with the substitution of Zr^4+^ by the larger Y^3+^ ions, which introduce lattice distortions and inhibit crystal growth during post-deposition annealing. Similar behaviour has been reported for yttria-stabilized zirconia prepared by various physical vapour deposition techniques [15,16]. The diffraction peaks originating from the ITO substrate also exhibit noticeable broadening after film deposition, particularly for the Y5 sample (Figure 1, inset). The crystallite sizes of the monoclinic and orthorhombic ZrO_2_ phases were calculated from their isolated diffraction peaks. In contrast, the characteristic reflections of tetragonal Y-stabilized zirconia (JCPDS card No. 98-005-9166) overlap with the main diffraction peaks of the ITO substrate, preventing reliable peak separation under the present experimental conditions. Therefore, the calculated crystallite size corresponds to the combined ITO/t-ZrO_2_ contribution rather than to the tetragonal phase alone. The apparent crystallite size associated with the ITO reflections decreases from 34.33 nm for the bare substrate to 24.58 nm for the Y5-coated sample (Table 1). The progressive broadening of these overlapping reflections with increasing yttrium content suggests an increasing contribution of the tetragonal zirconia phase, which is characterized by a smaller crystallite size, in agreement with previous reports [15,16,26].

The coexistence of monoclinic and tetragonal zirconia is further supported by the AFM observations discussed in the following section, which reveal significant modifications in grain morphology after yttrium incorporation. The progressive stabilization of the tetragonal phase with increasing yttrium content is consistent with the well-established role of Y^3+^ as a stabilizing dopant in zirconia. The substitution of Zr^4+^ by Y^3+^ generates oxygen vacancies for charge compensation, thereby lowering the free-energy difference between the monoclinic and tetragonal polymorphs and favouring the stabilization of the latter at room temperature [27].

In addition to the monoclinic reflections, the Y3 film exhibits two weak diffraction peaks located at 2θ = 31.88° and 34.38°, which can be indexed to orthorhombic zirconia (space group *Pnma*, JCPDS No. 98-003-8847). Although this phase is metastable and is generally observed under specific thermodynamic conditions [28,29], its presence suggests that the intermediate yttrium concentration promotes a transient structural rearrangement during crystallization. The average crystallite size of the orthorhombic phase (36.25 nm) is considerably larger than that of the monoclinic phase, indicating that this phase nucleates and grows through a different crystallization mechanism [27].

### 2.2. Atomic Force Microscopy

**Figure 2:**
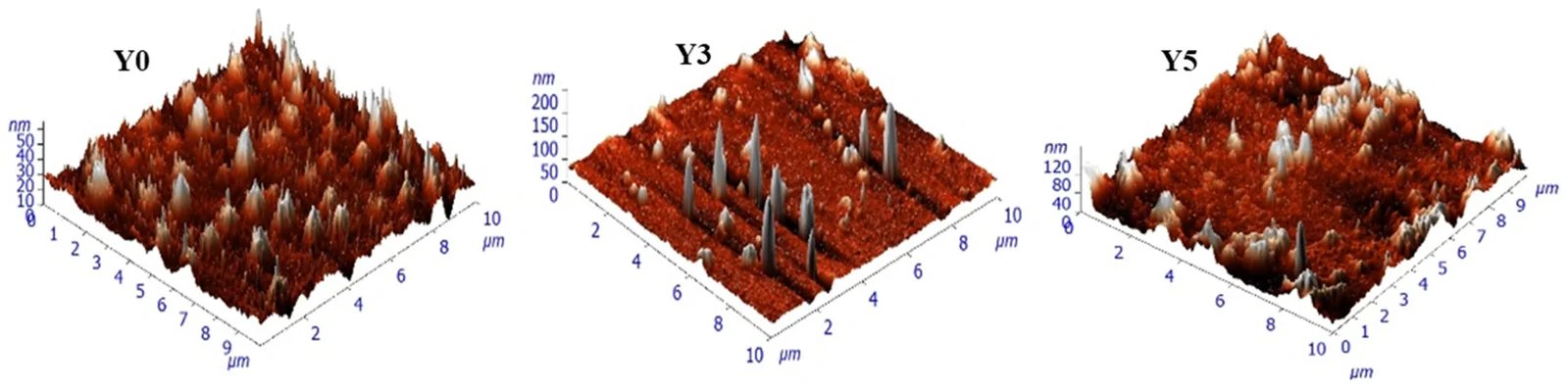
AFM 3D surface topography images of the Y0, Y3, and Y5 thin films.

The relationship between surface topography and photocatalytic properties is complex and is influenced by numerous physicochemical parameters [30]. The three-dimensional atomic force microscopy (AFM) images of the undoped (Y0) and yttria-doped (Y3 and Y5) ZrO_2_ thin films are presented in Figure 2. The AFM images reveal continuous and homogeneous surfaces for all films, indicating uniform film growth during reactive RF magnetron sputtering deposition. However, yttrium incorporation significantly modifies the surface topography and the growth morphology of the zirconia films. The grain size and surface roughness were evaluated over an area of 10 × 10 µm^2^.

The 3D topography image of the undoped ZrO_2_ film (Y0) revealed a granular morphology characterized by vertically oriented, nanosized columnar grains that were relatively homogeneously distributed across the surface. The root mean square roughness (R_RMS_) was 5.2 nm, which is higher than that reported for ZrO_2_ films deposited by the High-Power Impulse Magnetron Sputtering (HiPIMS) technique (R_RMS_ = 1.65 nm) [5,31,32].

The R_RMS_ roughness increased from 13.8 to 18.4 nm with increasing yttrium content. This increase is accompanied by grain growth and grain agglomeration, leading to a transition of the surface morphology from a columnar to a cluster-like structure. It can be supposed that one of the crystalline phases and/or a newly formed phase is preferentially oriented relative to the substrate plane. This behaviour may indicate that one of the crystalline phases, and/or a newly formed phase, preferentially grows with an orientation parallel to the film–substrate interface [33].

The observed morphological evolution is consistent with the structural changes identified by XRD. The stabilization of the tetragonal zirconia phase, together with the reduction in monoclinic crystallite size, indicates that yttrium modifies both the crystallization process and the surface diffusion kinetics during film growth. The incorporation of Y^3+^ ions into the ZrO_2_ lattice generates local lattice distortions and oxygen vacancies, which influence the nucleation and growth process and consequently influence the final surface morphology.

The AFM observations further suggest that the increase in yttrium concentration modifies not only the average surface roughness but also the spatial distribution of the surface features. Such changes are expected to influence the effective surface area and the density of catalytically active sites. Nevertheless, the photocatalytic performance cannot be explained solely by morphological effects, indicating that the surface chemical composition and defect structure, discussed in the following section, will provide additional information in determining the photocatalytic activity of the yttria-doped zirconia films.

### 2.3. X-Ray Photoelectron Spectroscopy Analysis

**Figure 3:**
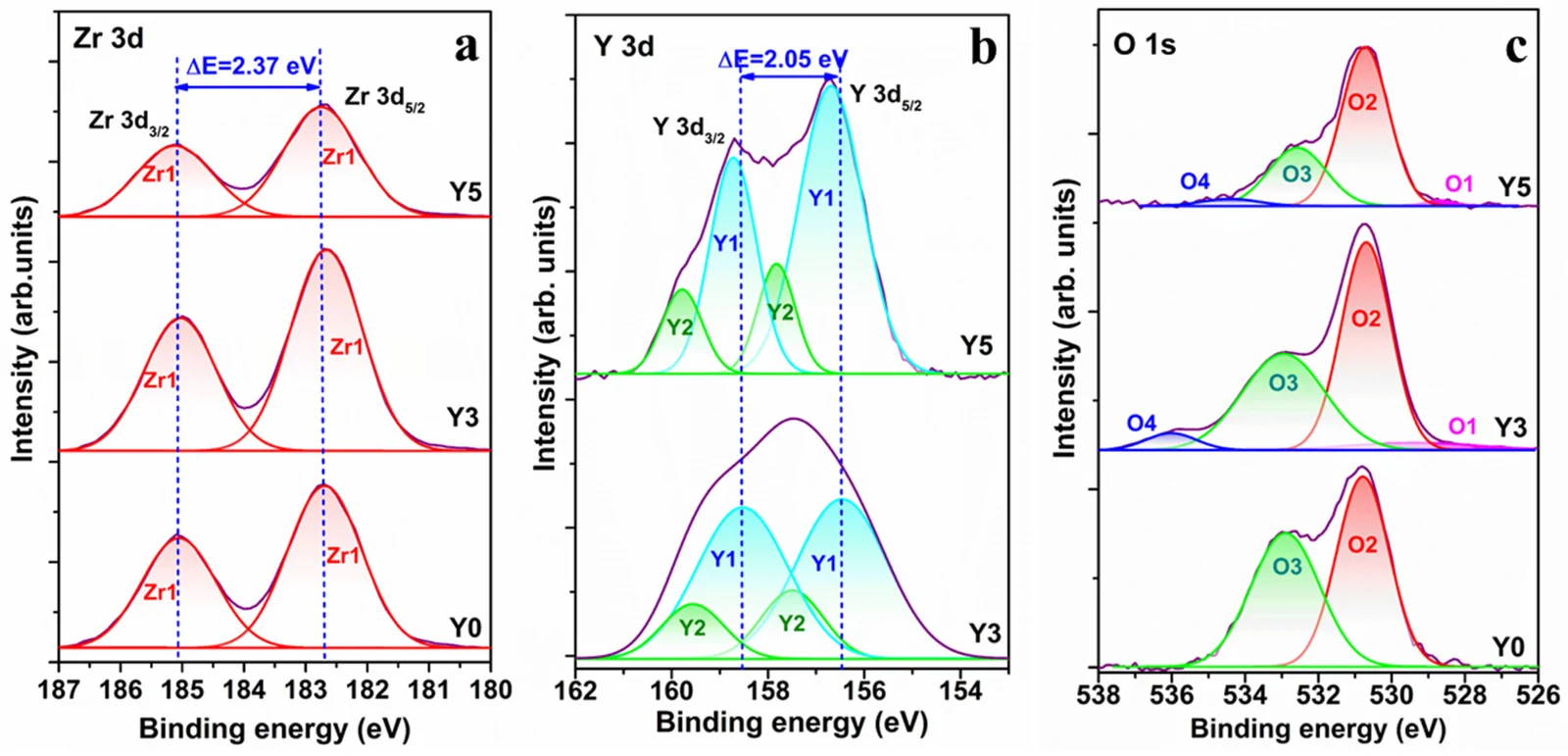
High-resolution XPS spectra and peak fitting of (**a**) Zr 3d, (**b**) Y 3d and (**c**) O1s for Y0, Y3 and Y5 films.

The electronic states of the constituent elements in the undoped (Y0) and yttria-doped (Y3 and Y5) zirconia films were investigated by X-ray photoelectron spectroscopy (XPS). The high-resolution spectra of Zr 3d, Y 3d and O 1s are presented in Figure 3, providing insight into the effect of yttrium incorporation on the local chemical environment and defect chemistry of the films.

The high-resolution Zr 3d spectra (Figure 3a) were fitted using a single symmetric doublet corresponding to the spin-orbit split Zr 3d_5/2_ and Zr 3d_3/2_ components, separated by 2.37 eV, in good agreement with the literature [34]. This result indicates that zirconium is present predominantly in the Zr^4+^ oxidation state and bonded to lattice oxygen (Zr-O_latt_, Zr1 component). The Zr 3d_5/2_ core level exhibits binding energies at 182.69 eV (Y0), 182.77 eV (Y3) and 182.75 eV (Y5), respectively, which are very close to the Zr 3d_5/2_ binding energy of tetragonal films (182.9 eV) [35]. The slight shift of binding energy to higher values may suggest that yttrium strengthens the tetragonal phase; the smaller yttrium concentration (0.27% (Y3) and 0.90% (Y5) atomic percentages) is likely to have a smaller effect on the Zr 3d binding energy.

The high-resolution Y 3d XPS spectra were deconvoluted into four peaks (Figure 3b). The peaks located at 156.46 eV and 158.49 eV for Y3, and 156.69 eV and 158.72 eV for Y5, were assigned to Y 3d_5/2_-O and Y 3d_3/2_-O bonds (Y1 peaks, Figure 3b), respectively, confirming the incorporation of Y^3+^ ions into the zirconia lattice [20]. Additional higher-binding-energy satellite components, located at 157.49 eV and 159.56 eV for Y3 and at 157.82 eV and 159.78 eV for Y5, were attributed to Y 3d_5/2_ and Y 3d_3/2_ states associated with oxygen vacancies (V_O_) Y^3+^-V_O_ and hydroxide groups Y^3+^- OH bonds (Y2 peaks) [34]. The presence of these satellite peaks, in agreement with previous reports [20,34], can be associated with Y^3+^ tendency to segregate to the Y-doped ZrO_2_ (tetragonal) film surface, which increases the surface oxygen vacancies neighbouring yttrium atoms (19% for Y3 and 21% for Y5), associated defects, and interaction with air humidity. These help to improve the photocatalytic properties of doped ZrO_2_ films.

The high-resolution O 1s spectra of the undoped ZrO_2_ and yttria-doped ZrO_2_ films were fitted into two components for the Y0 film and four components for the Y3 and Y5 films (Figure 3c). The low-binding-energy O1 component, located at 529.39 eV (4.70% contribution) for Y3 and 528.69 eV (1.20% contribution) for Y5, was assigned to adsorbed dioxygen (Zr,Y)-O_2_ bonds on the film surface. The O2 component, centred at 530.74 ± 0.04 eV, with relative contributions of 53% for Y0, 52% for Y3 and 66% for Y5, was assigned to lattice oxygen. The O3 component, located at 532.89 eV for Y0, 532.94 eV for Y3 and 532.57 eV for Y5, was ascribed to oxygen-vacancy (V_O_) and dissociated water species (mainly HO groups) [36]. The O4 component, observed only for the yttria-doped films at 536.02 eV (4.5%) for Y3 and 534.40 eV (3.50%) for Y5, was attributed to adsorbed water molecules from air humidity. The total contribution of O1, O3 and O4 bonds for Y doped zirconia decreases from 49.20% for Y3 to 34.70% for Y5, with increasing yttrium content. The results clearly show that, despite the lower roughness of the Y3 film compared to Y5, the higher contribution of oxygen vacancies (partially occupied by adsorbed water species) and matrix defects will act as scattering centres. This prevents the recombination of electrons and holes, thereby enhancing photocatalytic activity.

The slightly higher Y 3d satellite contribution observed for Y5 (approximately 2%) can be associated with its higher Y concentration. In contrast, the higher defect-related contribution observed in the O 1s spectrum of Y3 may be related to its three-phase structure (monoclinic, tetragonal, and orthorhombic), which provides additional phase interfaces and associated defects compared with Y5, where only monoclinic and tetragonal phases are present.

The combined XRD and XPS results demonstrate that yttrium incorporation not only stabilizes the tetragonal phase but also modifies the surface chemistry of the ZrO_2_ films. Oxygen vacancies and zirconia matrix defects act as trapping centres for photogenerated charge carriers, thereby suppressing electron–hole recombination, while surface hydroxyl groups provide active sites for the adsorption and activation of reactant molecules. These synergistic effects promote the generation of reactive oxygen species and account for the enhanced photocatalytic activity of the yttria-doped films.

### 2.4. Optical Properties Analysis

**Figure 4:**
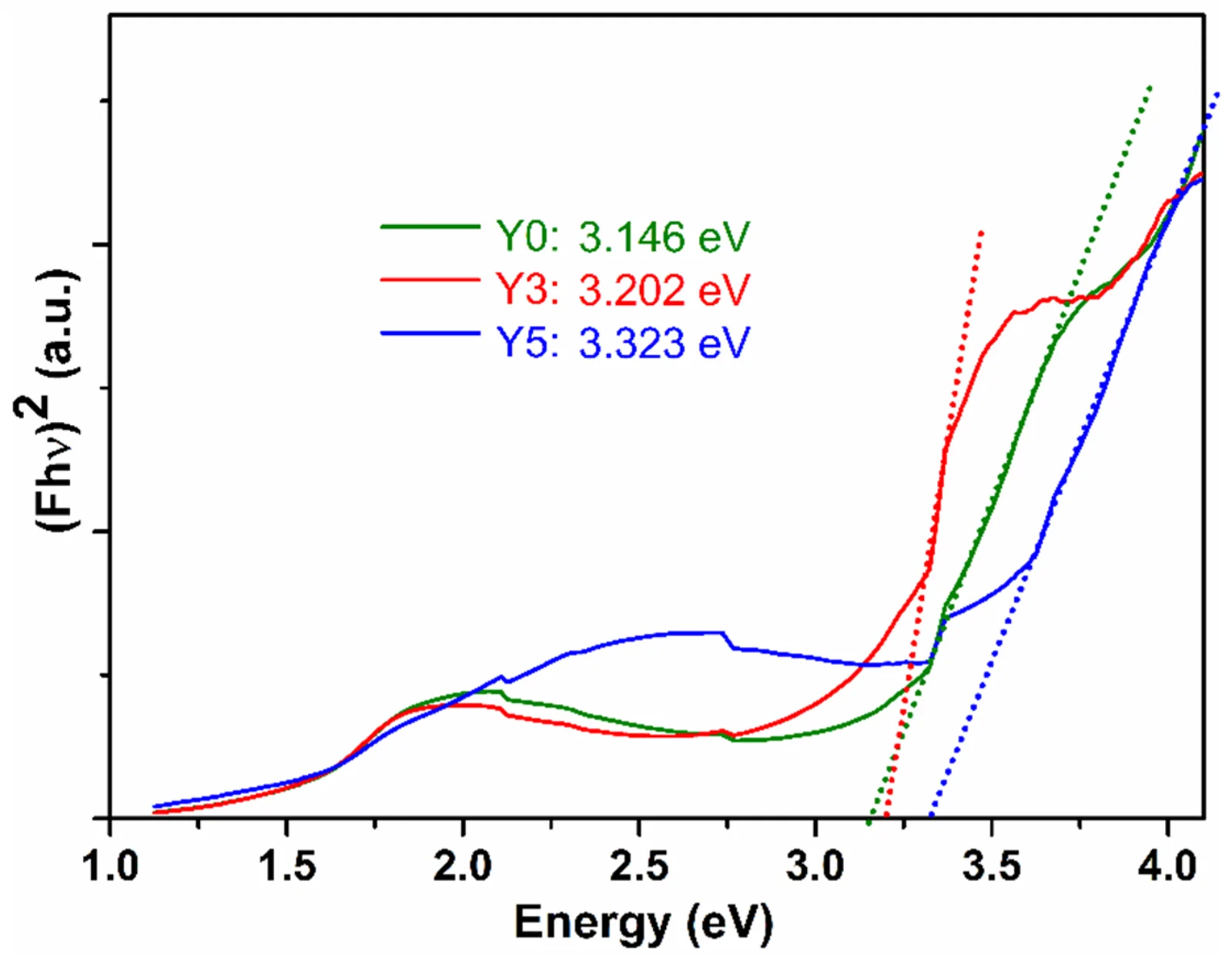
The dependences (Fhν)^2^$=f\left(h\nu \right)$ for the investigated films.

Optical properties are important in evaluating the efficiency of the catalyst interaction with electromagnetic radiation. Thus, by analyzing the optical bandgap (E_g_) obtained from optical absorption spectra, it can be observed that yttrium doping leads to a slight increase in the bandgap, from 3.146 eV (Y0) to 3.202 eV (Y3) and 3.323 eV (Y5) (Figure 4), with the films remaining UV-absorbent (394–373 nm). Thus, yttrium doping of ZrO_2_ does not lead to the desired photocatalytic property of maximizing solar light utilization. The obtained values are relatively low compared with the reported values for spin-coated films, which range from 4.11 to 4.15 eV. This difference can be related to the deposition method and doping concentration, which play significant roles [37,38]. The lower Eg values compared with those reported in the literature could be attributed to the dopant size, which induces modifications in the material’s density of states and the splitting of the valence and conduction bands into discrete electronic levels [39]. This suggests that the photocatalytic behaviour is influenced not only by the variation in bandgap but also by other factors, such as modifications of the electronic structure, the formation of oxygen-vacancy-related defects, and changes in phase composition, which may affect charge-carrier separation and, consequently, the photocatalytic activity.

Here, we have continued the investigation begun in the previous work [5,40], related to the possible coupling of the two semiconductors (film-substrate), due to the proper edges of the conduction band (CB) and the valence band (VB). There are papers that take into consideration values from the literature for the mentioned pair [41,42]. But, as we have previously studied [5,40], the band edges are dependent on many specific factors and need to be calculated for each particular case. We have previously observed that the ITO substrate “helps” the photocatalytic process for the ZrO_2_ films doped with nitrogen, since there is a charge separation that decreases the recombination rate of the electrons and holes.

By using the values of the bandgap *E_g_*, the energies of the valence band (*E_VB_*) and conduction band (*E_CB_*) were calculated by applying the atom’s Mulliken electronegativity (*χ*) [5,43]:

(2) $$E_{CB}=\chi -E_{C}+0.5E_{g}$$

(3) $$E_{VB}=E_{CB}+E_{g}$$

where *Ec* is the energy of free electrons (4.5 eV) and *χ* is the absolute electronegativity of the film [44]. *χ* was found to be 5.915 eV, 5.910 eV and 5.895 eV for Y0, Y3 and Y5, respectively. The molar composition of the samples and the corresponding χ values were calculated from the XPS data using the charge neutrality rule. The values of the *E_VB_* and *E_CB_*, calculated with Equations (2) and (3), are listed in Table 2. The rise of the valence band edge can be attributed to the presence of oxygen vacancies, leading to a slight increase in the O 2s orbital contribution to O^2−^ hybridization (increasing electronegativity), which induced new valence bands above the *E_VB_* of pristine ZrO_2_. In contrast, the decrease in *E_CB_* values is generated by the defect state levels created by the lower ionic charge of yttrium and the larger ionic radius of Y^3+^ ions. Thus, the bandgap increased due to the increase in Y concentration.

**Figure 5:**
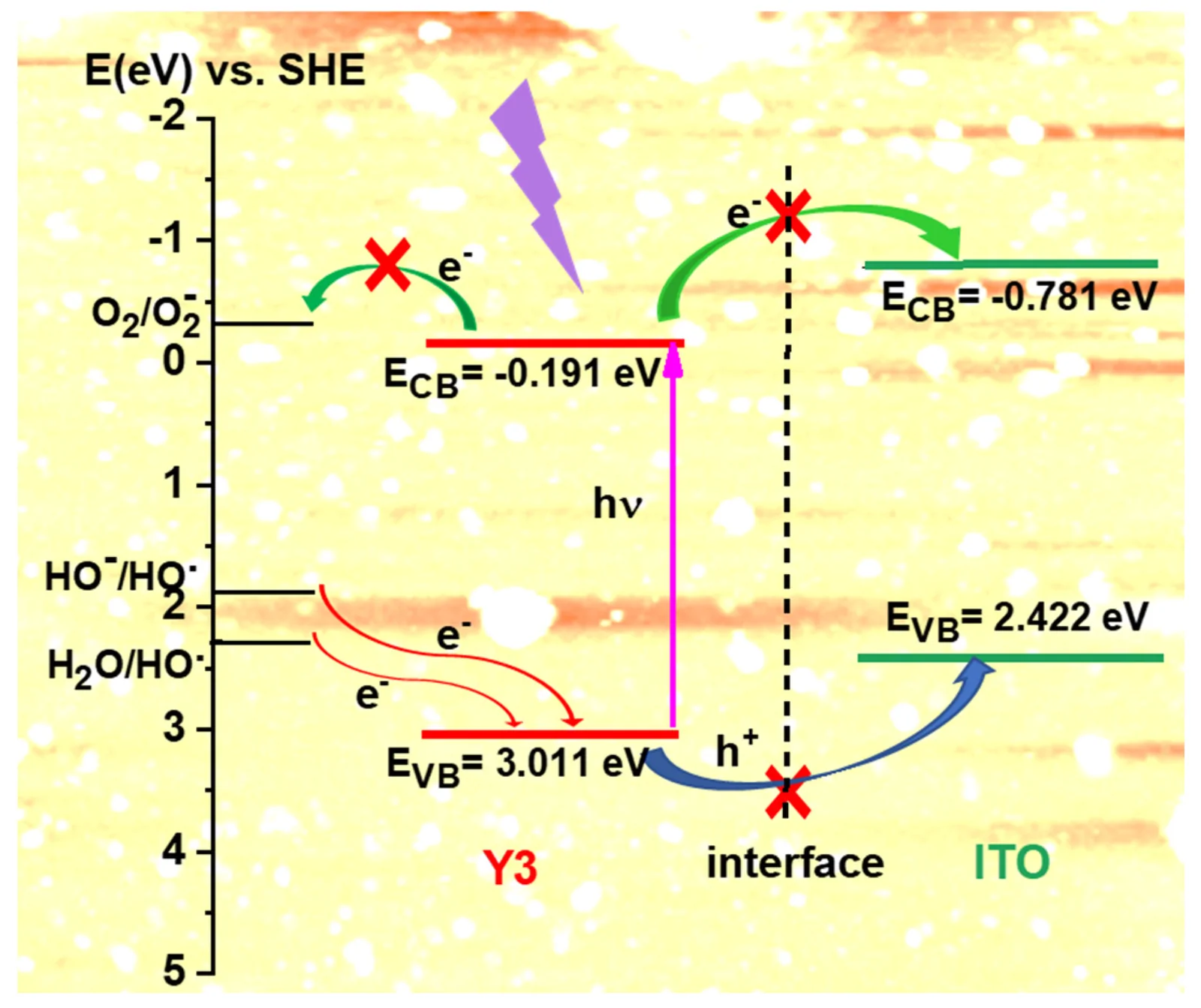
Possible charge transfer at the donor/acceptor molecule–film and film–substrate interfaces for the Y3 thin film.

We are interested in the feasible charge transfer between the studied films and the adsorbed (donors/acceptors) molecules. The redox potential of O_2_/O_2_^−^ is −0.33 eV vs. SHE [45], which is more negative than the *E_CB_* values for all the investigated films, as illustrated in Figure 5 for Y3. In fact, this means that the photoexcited electrons cannot reduce the adsorbed dioxygen, and the necessary superoxide does not form to participate in the direct oxidation of the adsorbed pollutant molecules on the films’ surface.

However, for the undoped ZrO_2_ films (UDZ) obtained by HiPIMS, the photoexcited electrons do reach the conduction band. An experimental comparison between the bandgaps of tetragonal and monoclinic ZrO_2_ thin films found a difference of up to 1 eV in the monoclinic film’s bandgap [35,46]. The XRD analysis of UDZ confirms the contribution of the monoclinic phase. Additionally, the optical bandgap, and implicitly the *E_CB_* and *E_VB_* values, can be affected by the defects and grain boundary stress due to the larger ionic size of Y^3+^ and the abundance of oxygen vacancies. The decrease in crystallite size (or increasing grain boundaries) widens the bandgap of Y3 and Y5 [47].

But looking at the redox potential of the donor molecules HO^−^/HO^•^ and H_2_O/HO^•^, their potential levels (1.89 eV and 2.27 eV, respectively [5,48]) are more negative than the *E_VB_* of Y0, Y3 and Y5, so they can donate electrons to the holes from the valence bands of the studied films. Therefore, holes act as the main driving force in this process by oxidizing adsorbed water into hydroxyl radicals or directly decomposing the dye molecules.

From Figure 4, it can be observed that in the studied films, it is not possible for either the holes (h^+^) or the electrons to be transferred to the ITO substrate. This is because their *E_VB_* and *E_CB_* values are more positive than those of ITO. So, we conclude that for all the investigated films, the ITO substrate does not contribute to charge separation, not even for the undoped ZrO_2_, obtained in this case with the RF sputtering method. So, their good photocatalytic performance cannot be related to the ITO substrate as in the case of previously studied ZrO_2_ films doped with nitrogen, obtained by HiPIMS. It is known that the high-power impulse magnetron sputtering deposition method offers more uniform films with a smaller active surface area and a lower number of active centres, respectively.

### 2.5. Hydrophilic Properties

**Figure 6:**
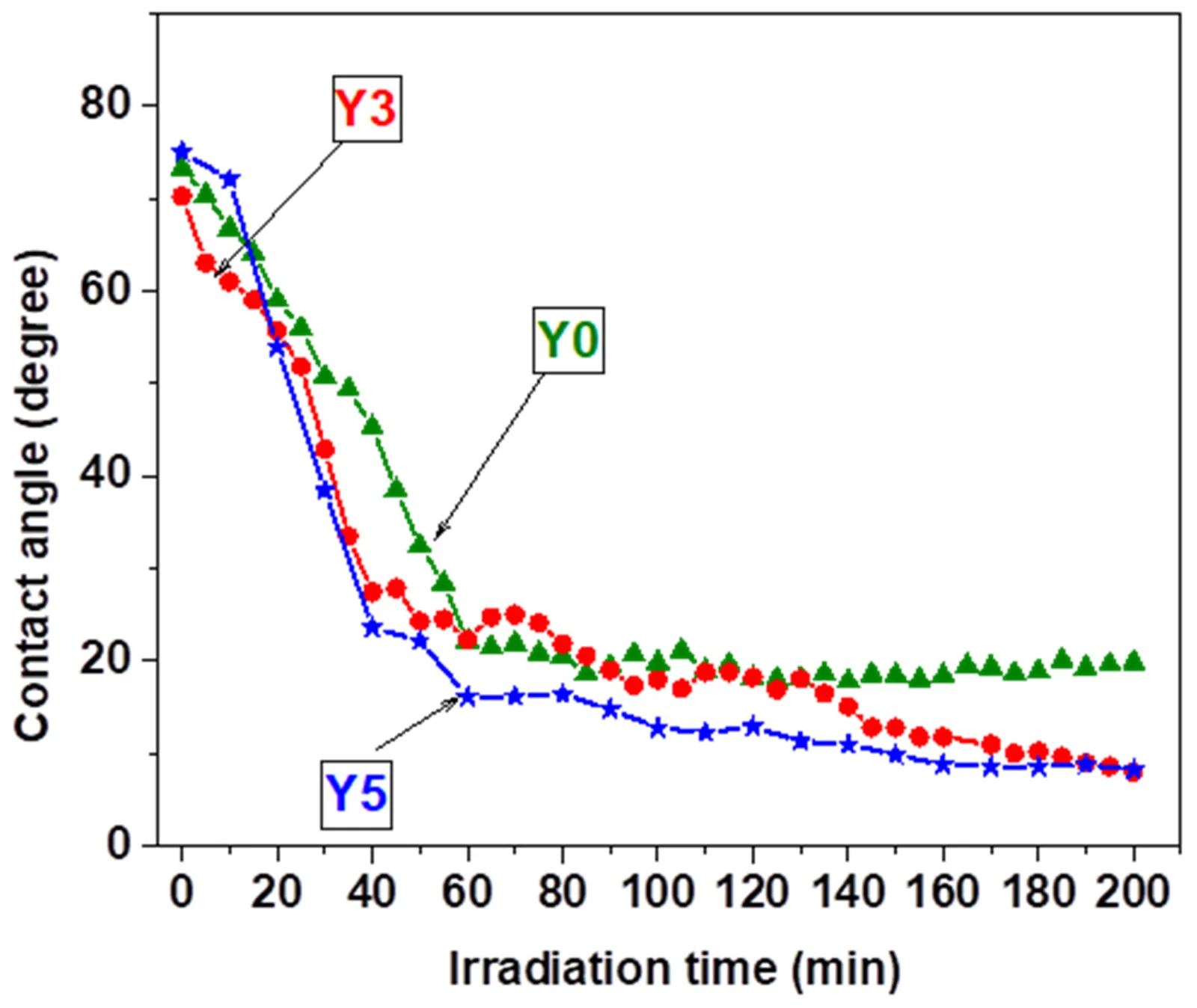
Time dependence of the contact angle between deionized water and the surface of the studied films during UV irradiation.

Hydrophilicity measurements conducted on the analyzed thin films reveal enhanced wettability resulting from yttrium doping (Figure 6). While the evolution of the contact angle as a function of irradiation time follows a similar trend across all samples, one can see that the yttrium-doped films exhibit superior hydrophilic performance. During the initial 60 min of irradiation, the contact angle decrease rate for the doped specimens was 0.97 deg/min (Y3) and 1.10 deg/min (Y5), outperforming the 0.84 deg/min rate observed for the undoped film. Following this initial phase, a gradual decline occurs; after 200 min, the doped films achieve superhydrophilicity, reaching contact angles of 9 deg. [49], whereas the undoped film, Y0, reaches saturation at only 20 deg.

The enhanced hydrophilicity observed for the Y-doped films may be related to the structural and surface modifications induced by Y^3+^ incorporation into the ZrO_2_ lattice. The substitution of Zr^4+^ by Y^3+^ is accompanied by the formation of oxygen vacancies for charge compensation. Previous theoretical studies on YSZ have shown that water can interact strongly with the YSZ surface and that, in defect-containing surface regions, water may undergo dissociative adsorption, leading to the formation of surface hydroxyl species [50]. Such surface hydroxylation may favour the interaction of the film with water and, consequently, contribute to the enhanced wettability observed for the Y-doped samples. In addition, the Y-induced changes in phase composition, crystallite size and surface morphology may also affect the surface energy and wettability of the films.

**Figure 7:**
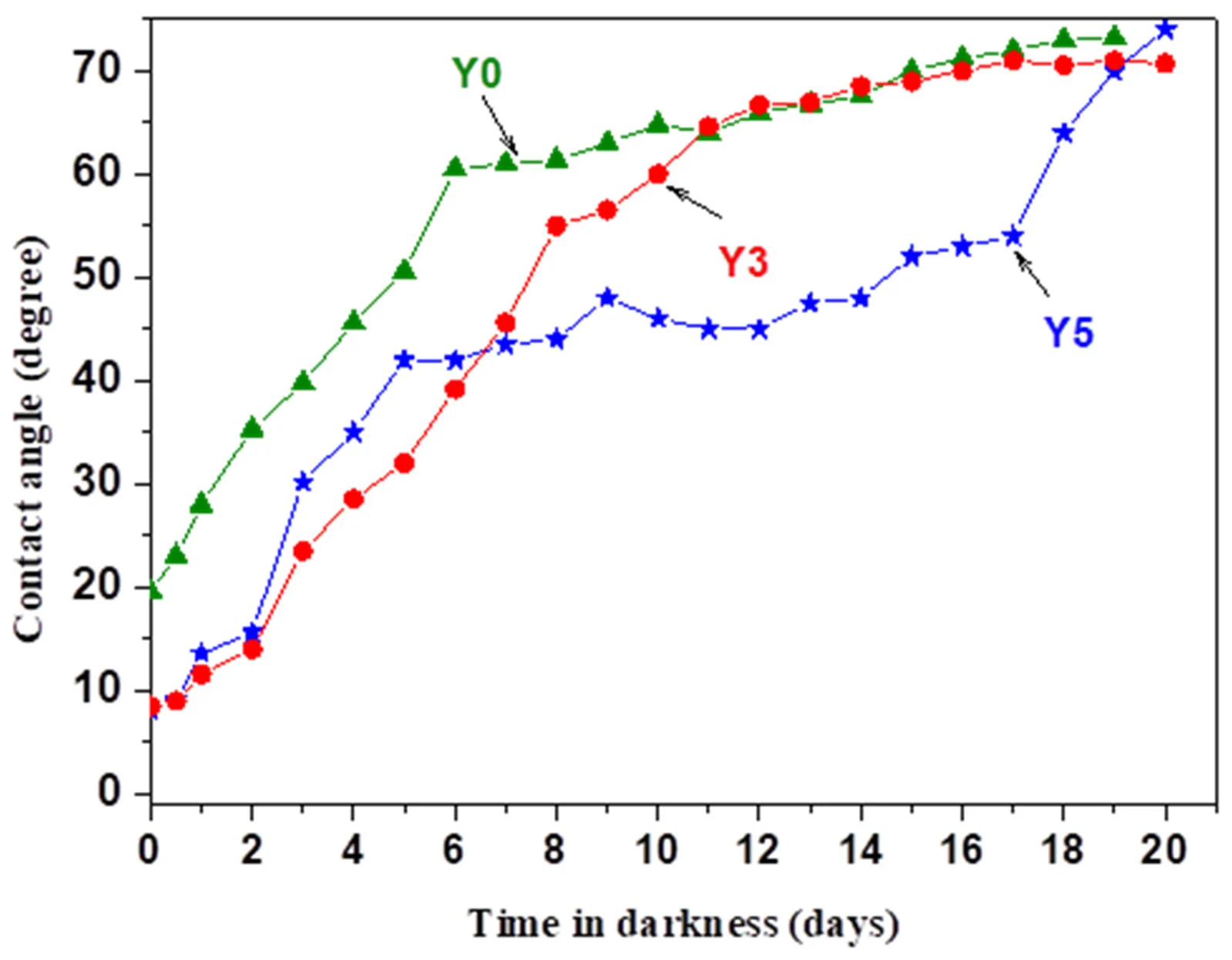
Time dependence of the contact angle between deionized water and the surface of studied films during deactivation.

In the next stage, we investigated the time required for the contact angle to return to its pre-irradiation values by keeping the thin films in the dark. The evaluation was performed over 20 days, during which the contact angles successfully reached their initial levels (Figure 7).

As shown in Figure 7, the deactivation speed is higher during the first 7–8 days (6.43 deg/day for Y5, 5.96 deg/day for Y3, and 7.18 deg/day for Y0). In the following days, the contact angles return more slowly to their original values (1.07 deg/day for Y5; 3.15 deg/day for the first segment and 0.67 deg/day for the second segment for Y3; and 0.94 deg/day and 6.60 deg/day for Y0).

Compared to the nitrogen-doped ZrO_2_ films previously studied, where the initial contact angle recovery time is less than 10 days [5,40], yttrium-doped ZrO_2_ exhibits a recovery time that is twice as long, making it highly recommended for self-cleaning applications.

### 2.6. Photocatalytic Activity and Mechanism of Dye Photocatalytic Degradation

We have explored the photocatalytic activity of the studied 10 × 20 mm films in the decomposition of RhB and MB dyes. Before evaluating the photocatalytic activity of the catalyst, the dye solutions and thin film systems were kept in the dark for 1 h to reach the adsorption–desorption equilibrium state. The Y0 film exhibits higher dye adsorption capacities (9.2% for MB and 8.40% for RhB), than the Y3 film (6.70% for MB and 7.70% for RhB) and the Y5 film (4.60% for both MB and RhB). The adsorption capacity is the consequence of electrostatic interactions between cationic dye molecules and the negative centres of the catalyst (adsorption) and donor-acceptor interactions between Lewis acid (Zr^4+^ and Y^3+^) and the donor atoms of RhB (O and N) and MB (N and S) (chemisorption). Based on the above values and the XPS analysis, it can be concluded that chemisorption is the dominant process through the oxygen vacancy active centres (48% for Y0 and 40% for Y3), followed by the surface roughness (higher R_RMS_ = 18.4 nm for Y5).

#### 2.6.1. Photodegradation of RhB and MB

Under UV irradiation, in the absence of a catalyst, the RhB and MB were photolyzed by less than 5% and 4%, respectively, for a 1 mg/L solution, while photolysis remained below 3% for the 3 and 5 mg/L solutions.

**Figure 8:**
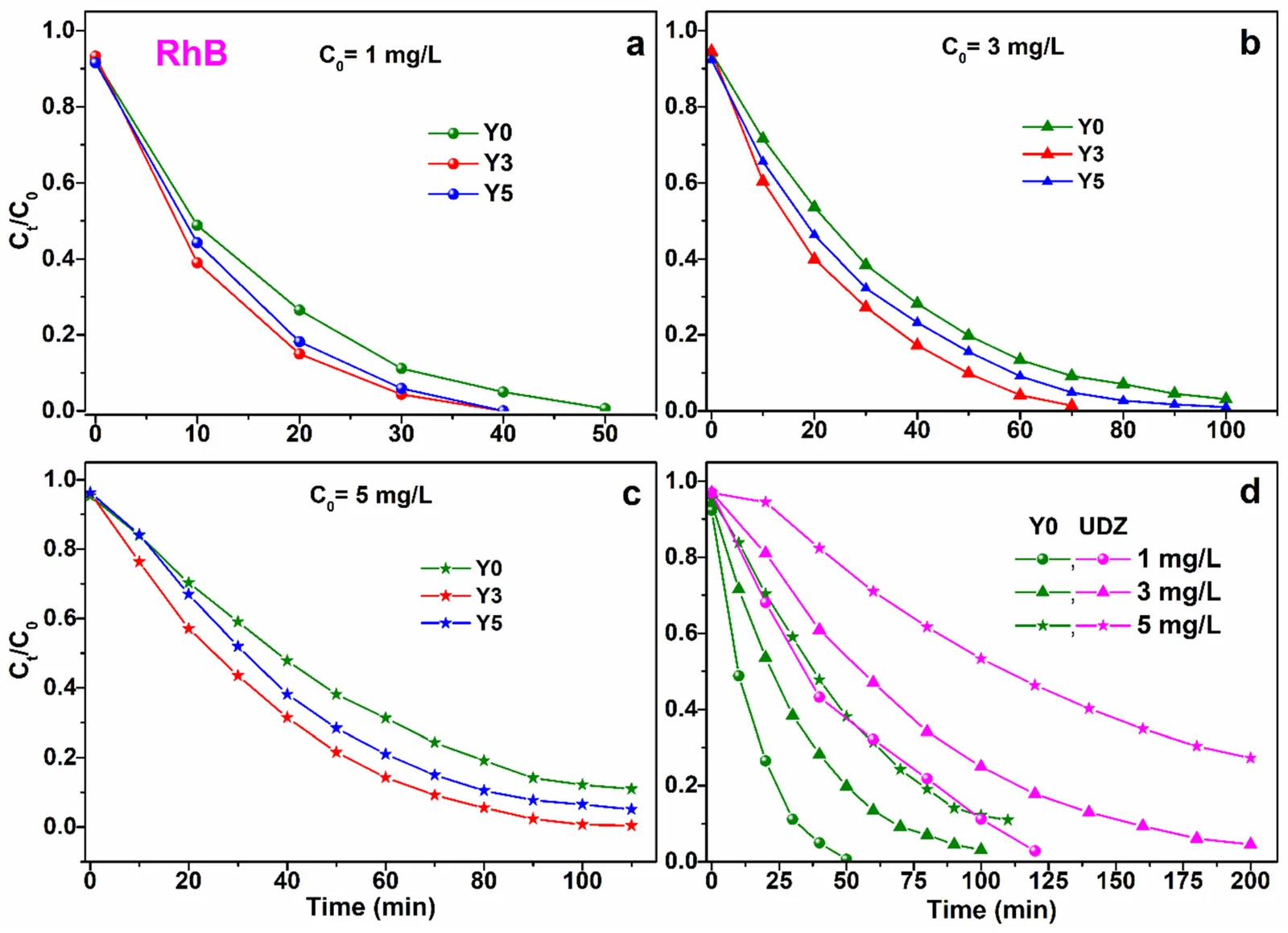
Comparison between the photodegradation efficiencies of Y0, Y3 and Y5 films for 1 mg/L (**a**), 3 mg/L (**b**) and 5 mg/L (**c**) RhB degradation, and Y0 with UDZ for all three RhB concentrations (**d**), under UV irradiation.

In order to evaluate the effect of dye concentration, comparative photodegradation experiments were carried out using RhB solutions with concentrations of 1 mg/L (S1), 3 mg/L (S3), and 5 mg/L (S5) over time on Y0, Y3 and Y5 films under UV light. As can be seen in Figure 8a–c, the degradation time increases proportionally with the solution concentration, while the degradation efficiency (DE) decreases as the solution concentration increases for all films. The highest DE values were registered for Y3: 99% (S1), 90.63% (S3) and 78.42% (S5) after 50 min of irradiation time (t_irr_), while Y0 has the lowest photocatalytic activity for S3 (79.74%) and S5 (31.91%).

In spite of this, the photocatalytic performance of Y0 is almost three times better than that of the ZrO_2_ film deposited by HiPIMS (UDZ) (Figure 8d). In our particular case, the lower contribution of the structural features (crystallite size, surface roughness) has a significant impact on the photocatalytic behaviour of the UDZ film.

Also, the DE differences between Y3 and Y5 catalysts increase with dye concentration, from 0% for S1 to ~8% for S5 at t_irr_ = 50 min. The lower degradation efficiency observed for the higher yttrium concentration may be due to the partial saturation or inactivation of active sites and the reduced ability of electrons and holes to separate.

**Figure 9:**
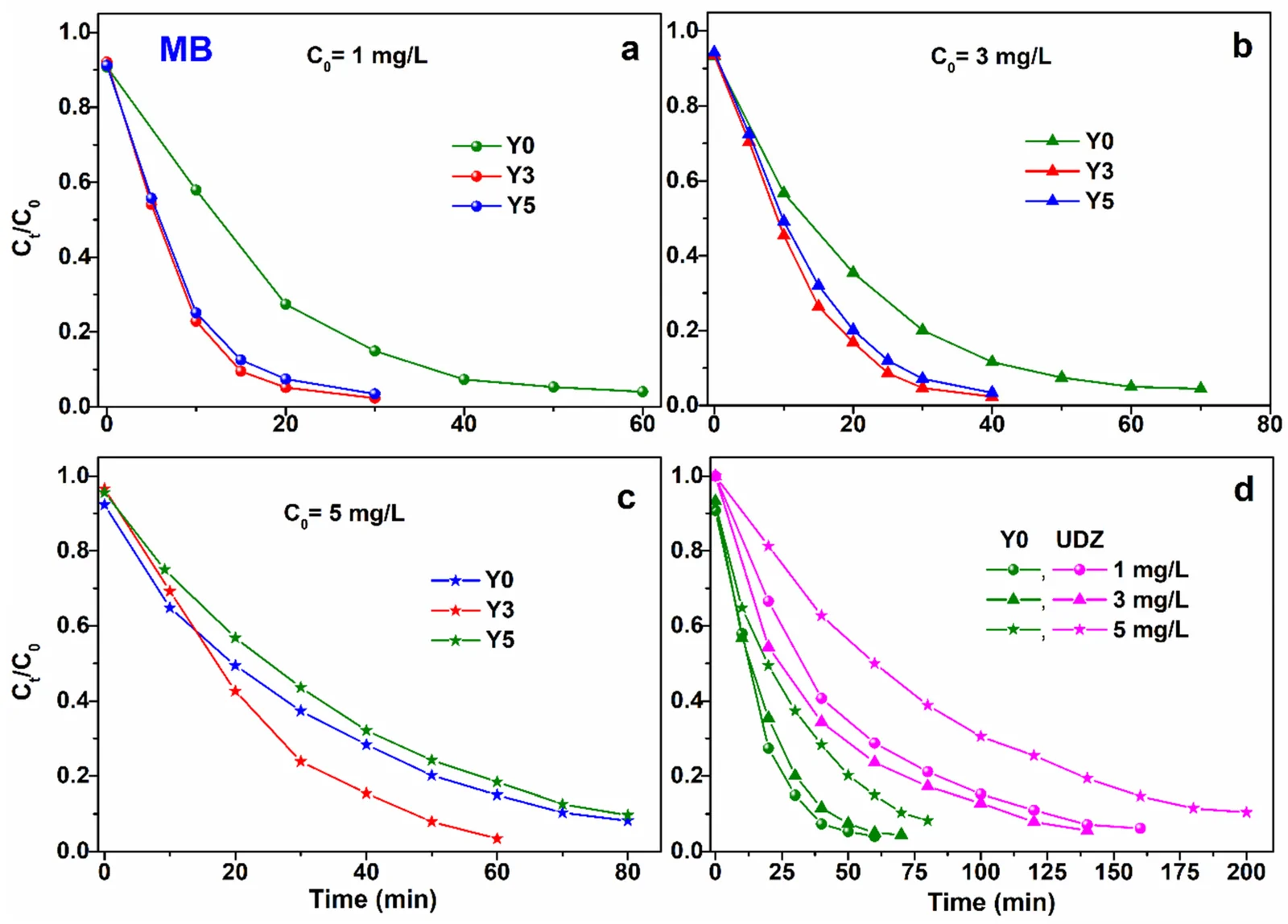
Comparison between the photodegradation efficiencies of Y0, Y3 and Y5 films for 1 mg/L (**a**), 3 mg/L (**b**) and 5 mg/L (**c**) MB degradation, and Y0 with UDZ for all three MB concentrations (**d**), under UV irradiation.

In Figure 9a–c, the different durations required for the photodegradation of RhB and MB are a consequence of the dyes’ structural and chemical composition particularities: (i) the smaller van der Waals molecular volume (0.261 nm^3^ [51]) than RhB (0.32 nm^3^ [52]), facilitating easier access to the photocatalyst active sites; (ii) electronic influence of the S and N atoms from the heterocyclic ring, which are more susceptible to interaction with oxidizing species; (iii) lower π-π resonance energy interaction, as reflected by its absorption maximum (λ_max_ = 663 nm; 15,083 cm^−1^) compared with RhB (λ_max_=553 nm; 18,083 cm^−1^).

Although the shape of the degradation curves is similar to that observed for RhB, the differences between the DE of the doped films are more pronounced. At relatively low MB concentrations (S1 and S3), the Y3 and Y5 films have very similar photocatalytic performance (ΔDE_Y3-Y5_ = 0% and 0.8%, respectively) after 40 min of irradiation time, whereas at the highest concentrations (S5), the DE values become much closer to that of Y0 (ΔDE_Y3-Y5_ = 12.28% and ΔDE_Y5-Y0_ = 4.10%). This behaviour could be explained by a high number of intermediate species, mainly organic derivatives and photo-generated oxygen reactive species (ROS), during the oxidation of a larger number of MB molecules in the first 20 min of irradiation (Figure 9c) [53]. These species may interact more strongly with the Y5 semiconductor surface, which can cause a redistribution of atoms on the crystallite’s surface [54], thus preventing the adsorption of other MB molecules on the active sites. Also, in the case of MB photodegradation, the Y0 film maintains superior photocatalytic performance compared with the UDZ film (Figure 9d).

Overall, according to this trend, the photocatalytic activity of the yttrium-doped thin films is superior to that of the undoped one. Notably, for both doped samples, the photodegradation efficiency reaches its maximum value of 100% after 40 min and 30 min of irradiation during the degradation of the S1 RhB and MB solutions, respectively.

#### 2.6.2. Photodegradation Mechanism

**Figure 10:**
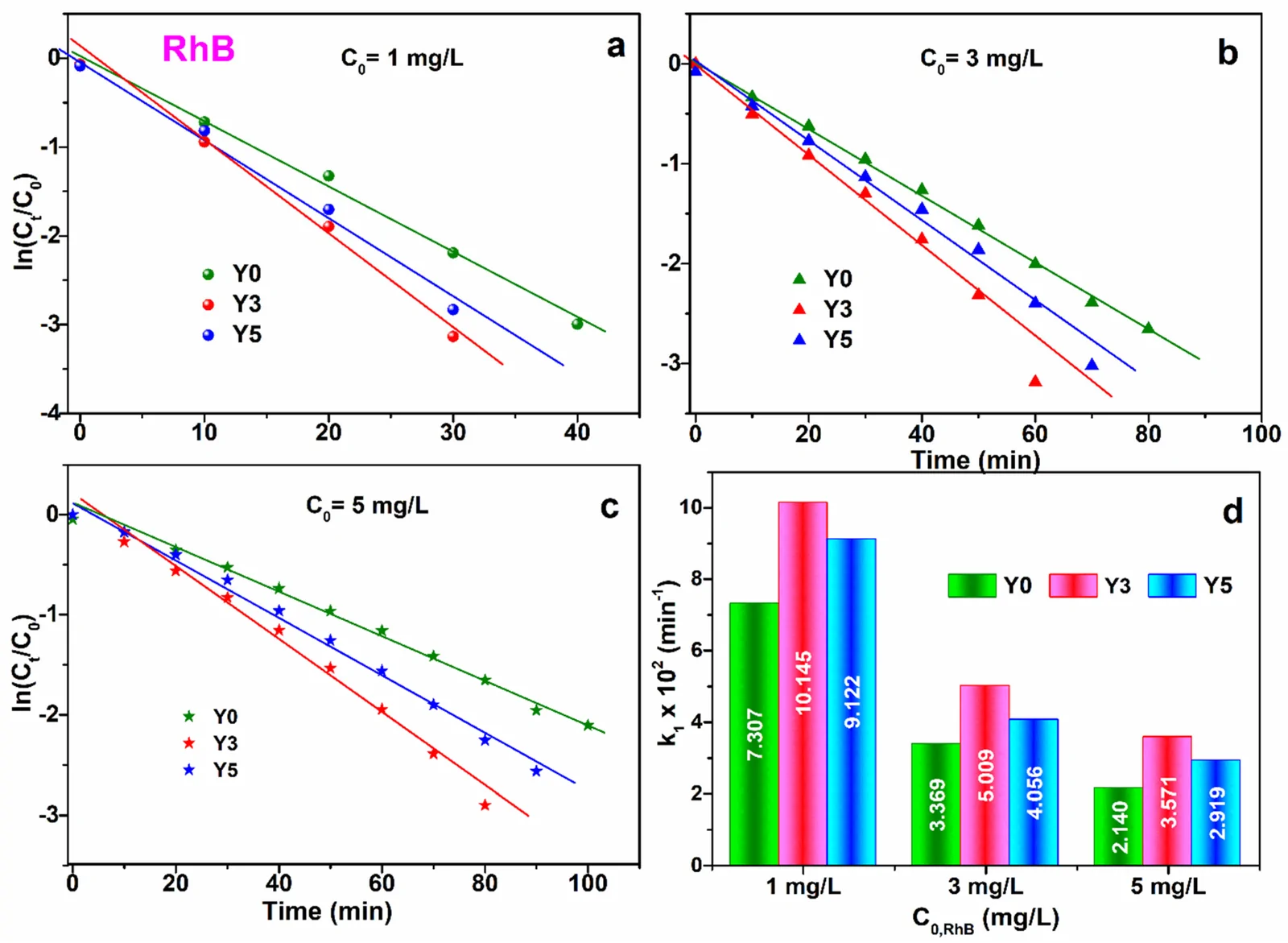
Kinetic plots for 1 mg/L (**a**), 3 mg/L (**b**) and 5 mg/L (**c**) RhB photodegradation on Y0, Y3 and Y5 films under UV light, and values of the apparent kinetic constant for the pseudo-first-order model (**d**).

**Figure 11:**
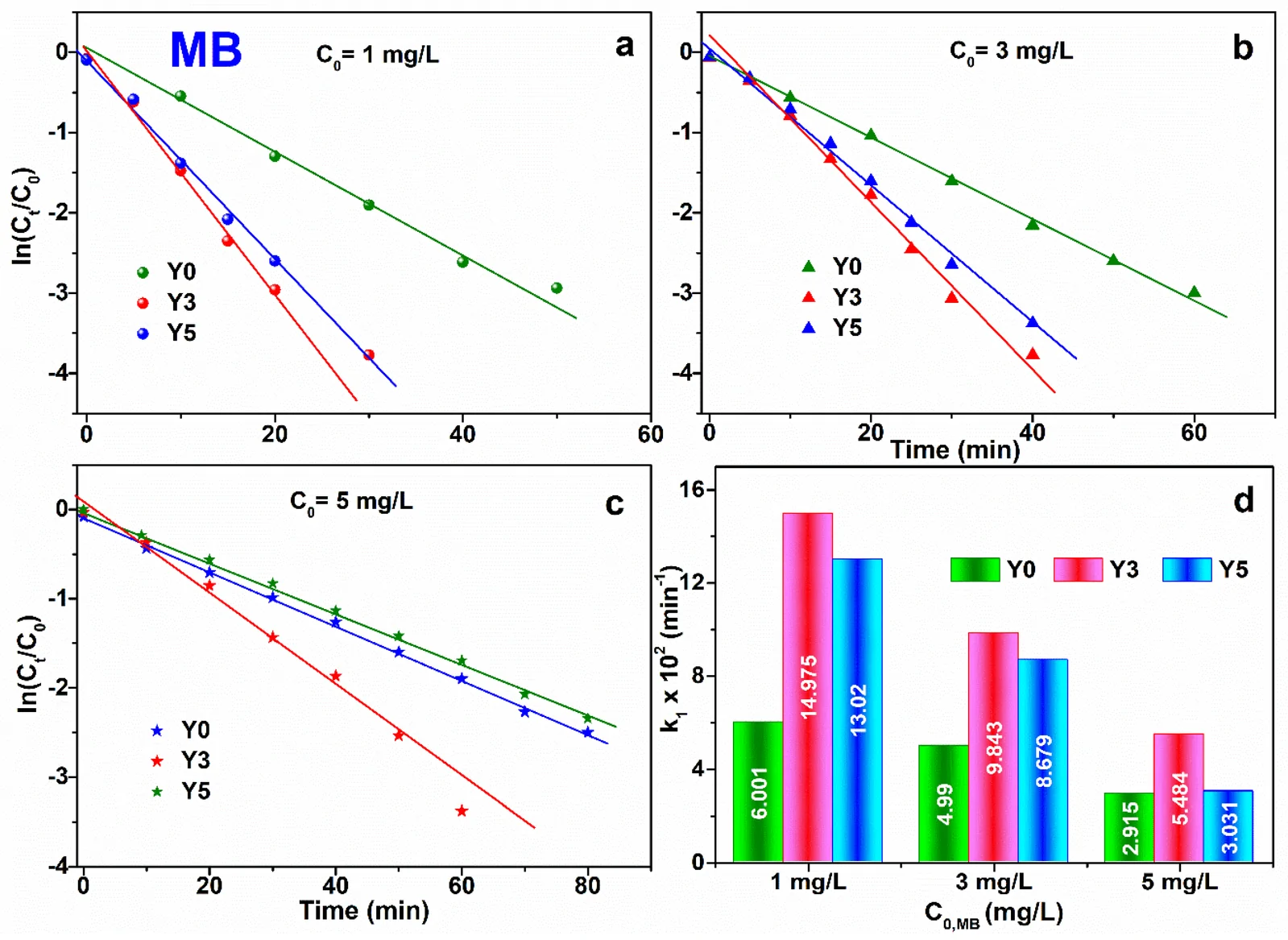
Kinetic plots for 1mg/L (**a**), 3 mg/L (**b**) and 5 mg/L (**c**) MB photodegradation on Y0, Y3 and Y5 films under UV light, and values of the apparent kinetic constant for the pseudo-first-order kinetic model (**d**).

Based on the above data, all reaction systems follow a first-order kinetic model. The apparent rate constants for RhB and MB photodegradation were determined from the linearization of ln(C_t_/C_0_) versus irradiation time using Equation (2) (Figure 10a–c and Figure 11a–c). The apparent pseudo-first order rate constants (k_1_) obtained from the linear fits are presented in Figure 10d for RhB and Figure 11d for MB.

Comparison of the k_1_ values reveals relatively high reaction rates for the entire set of undoped and Y-doped samples across all solutions. In the case of MB degradation, the reaction rates for the Y-doped films are higher for the same sample and solution compared to RhB degradation. Also, the reaction rate constants for the same solution concentration are 1.48 (S1), 1.96 (S3) and 2.14 (S5) times higher for the Y3 than for the Y5, in spite of the higher surface roughness of Y5. Among the photocatalysts, the Y0 film shows the lowest k_1_ values.

The adsorption of cationic RhB and MB takes place through interaction with HO^–^ on the photocatalyst surface:

(4) $$\left(Zr,Y\right)O_{2}-{HO}^{-}+Dye^{+}↔(Zr,Y)O_{2}-HO-Dye$$

Upon UV irradiation, an electron is ejected from the valence band to the conduction band, creating a hole in the valence band (Figure 4) [53]:

(5) $$\left(Zr,Y\right)O_{2}-HO-Dye+h\vartheta \to \left(Zr,Y\right)O_{2}(e_{CB}^{-}+h_{VB}^{+})-HO-Dye$$

The photogenerated electron–hole pairs migrate to the semiconductor surface. The holes oxidize the water:

(6) $$H_{2}O+h_{VB}^{+}\to H_{2}O^{+}\to HO^{•}+H^{+}$$

Furthermore, the hole can react with HO^−^ generated on the film surface to form HO^•^:

(7) $${HO}^{-}+h_{VB}^{+}\to HO^{•}$$

The generated hydroxyl radicals oxidize the organic molecules to form intermediate products, which are subsequently oxidized to CO_2_ and H_2_O:

(8) $$HO^{•}+Dye\;\to intermediary\;products\;\to CO_{2}+H_{2}O$$

In addition, the holes can react directly with dye molecules adsorbed on the photocatalyst:

(9) $$Dye+h_{VB}^{+}\to intermediary\;product$$

In our thin-film samples, the adsorbed dioxygen on the photocatalyst surface could not be directly reduced by electrons from the conduction band. The conduction band edge is more positive than the standard reduction potential of the O_2_/O_2_^−^ (ε^0^ = −0.33 eV) (Figure 4). In this case, electrons from the conduction band react with RhB and MB molecules:

(10) $$Dye+e_{CB}^{-}\to Dye^{•-}$$

Some of the photogenerated electrons are trapped by oxygen vacancies. They react with hydronium ions generated by the autoionization of water molecules and partial hydrolysis of the dye salts (pH < 6.5) [55]:

(11) $$H_{3}O^{+}+e^{-}\to H_{3}O^{•}$$

(12) $$H_{3}O^{•}\to H_{2}O+H^{•}$$

Both radicals can react with adsorbed O_2_, dye molecules and intermediates from photodegradation processes:

(13) $$O_{2}+H^{•}\to HO_{2}^{•}$$

The hydro-superoxide radical (HO_2_^•^) is a highly reactive oxygen species that can generate H_2_O_2_:

(14) $$2HO_{2}^{•}\to H_{2}O_{2}+O_{2}$$

(15) $$H_{2}O_{2}+e_{CB}^{-}\to HO^{-}+HO^{•}$$

In the degradation of RhB and MB, the holes and the electrons trapped in the oxygen vacancies play an important role in the photocatalytic performance of ZrO_2_ and Y-doped ZrO_2_ films. The most efficient generation and utilization of these charge carriers was observed for Y3 under UV light compared with Y0 and Y5. Overall, these results confirm that Y doping and the RF magnetron reactive sputtering method modulate the crystalline and electronic structure, thereby enhancing the photocatalytic performance of ZrO_2_/ITO films with a high potential for practical applications in environmental remediation.

### 2.7. Stability and Reusability

**Figure 12:**
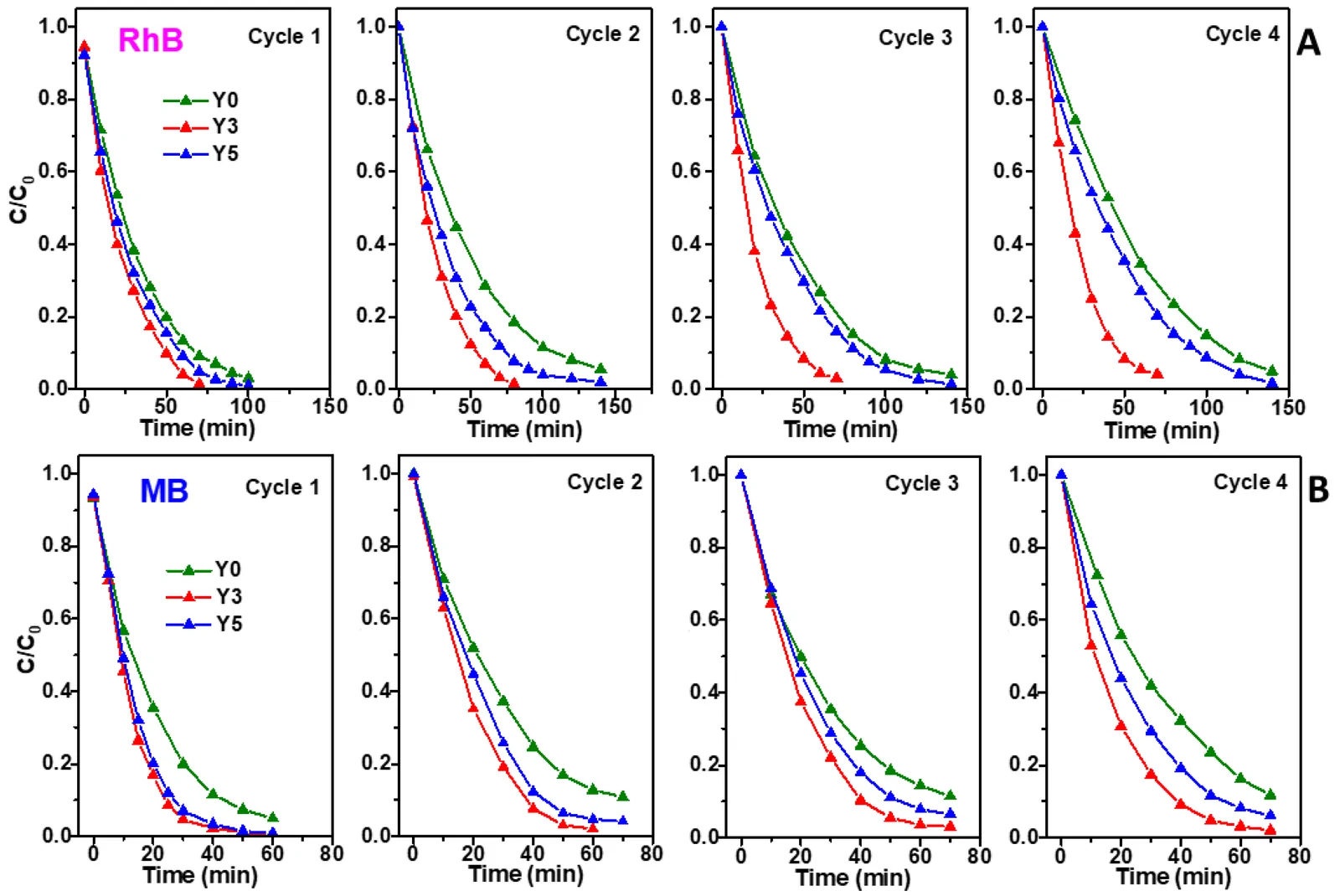
Photodegradation performance of the photocatalysts over four consecutive cycles for 3 mg/L RhB (**A**) and MB (**B**) solutions.

We investigated the stability and reusability of the films by examining the photodegradation performance of all three photocatalysts over four consecutive cycles for the 3 mg/L RhB solution (Figure 12).

One can observe that all samples exhibit high stability, with the lower-doped sample, Y3, showing the highest decomposition activity and the best stability. It is worth noting that the tetragonal phase is present in the yttrium-doped samples, which can induce a higher concentration of oxygen vacancies—beneficial for efficient photocatalysis, as observed for the previously studied low-nitrogen ZrO_2_ thin film [40]. Additionally, the presence of the orthorhombic ZrO_2_ phase, identified in the Y3 film, likely contributes positively to its superior degradation efficiency and stability. The Y5 sample initially shows behaviour similar to Y3, but its yield approaches that of the undoped sample Y0 by the final cycle, proving to be the least stable. In this case, the role of oxygen vacancies is entwined with the grain size effect, making it difficult to separate their individual contributions. Electrons captured in the oxygen-vacancy traps lead to the formation of ionized oxygen vacancies, below the energy level of V_O_, that can act as recombination centres for holes and electrons [17]. On the other hand, the higher Y^3+^ concentration in the Y5 ZrO_2_ matrix may disturb the lattice symmetry of the host, resulting in the redistribution of atoms within the crystallites and a decrease in the active surface [56].

The structural stability of ZrO_2_-based thin films under repeated photocatalytic cycles was investigated in our previous study [5], where XRD analysis performed after four consecutive photocatalytic cycles revealed that the crystal structure of the films remained essentially unchanged. In the present study, the stable photocatalytic performance observed over four consecutive degradation cycles, without any significant loss of activity, further supports the excellent stability and reusability of the investigated thin films.

**Figure 13:**
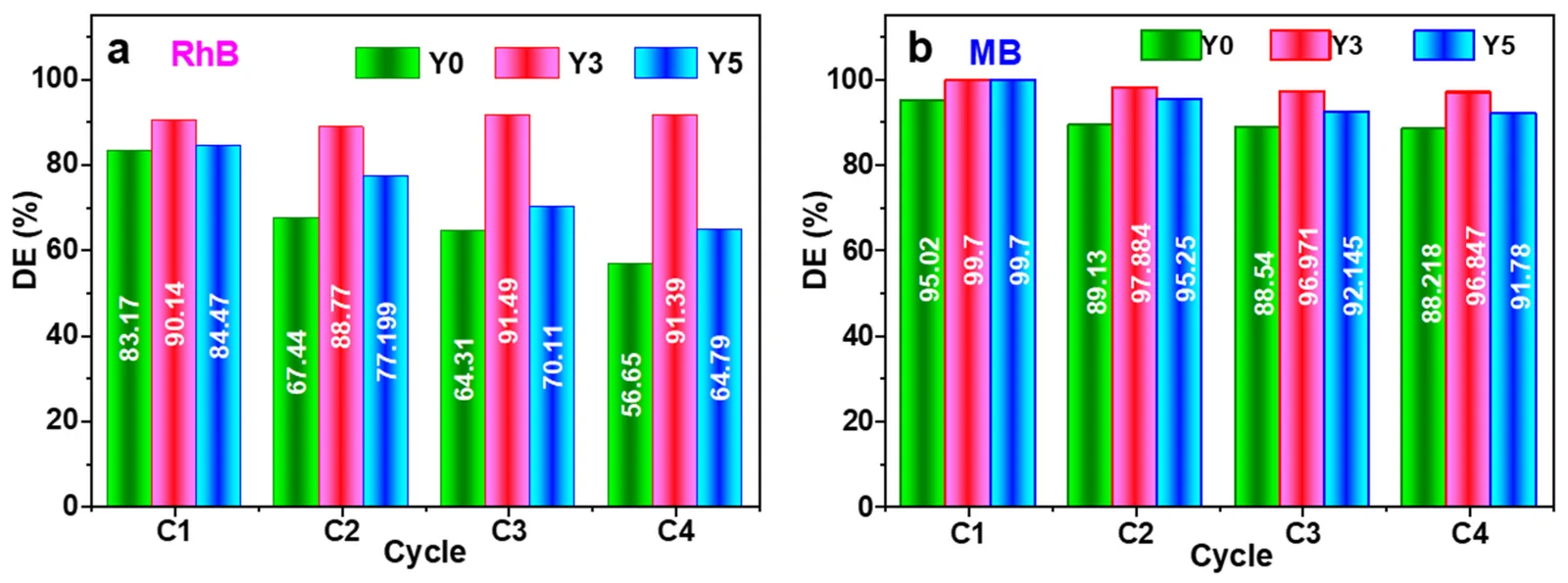
The degradation efficiency of the 3 mg/L RhB (**a**) and MB (**b**) solutions on the investigated films after 50 min of UV irradiation.

For a better comparison, Figure 13 presents the degradation efficiency of the RhB and MB solutions on the investigated films after 50 min of UV irradiation.

Photocatalytic studies on yttria-stabilized zirconia (YSZ) are still limited, since the majority of published works have focused on its structural and electrochemical properties rather than on photocatalytic applications. Consequently, the present results are compared with the limited literature available for YSZ as well as with representative studies on pure ZrO_2_ and cation-doped ZrO_2_ photocatalysts in order to evaluate the influence of yttrium doping on photocatalytic performance (Table 3).

As shown in Table 3, the photocatalytic performance of ZrO_2_-based materials strongly depends on several factors, including the crystalline phase, morphology, synthesis route, dopant type, and experimental conditions employed during photocatalytic testing. Direct comparison between different studies should therefore be made with caution, since parameters such as the initial dye concentration, catalyst loading, irradiation wavelength, light intensity, and exposure time may significantly influence the degradation efficiency [23,57,58].

The highest photocatalytic efficiencies reported for ZrO_2_-based materials are generally achieved using nanoparticle photocatalysts, with degradation efficiencies frequently exceeding 85–90% for methylene blue. Such enhanced performance is mainly attributed to the large specific surface area of nanoparticles, which provides a higher density of active sites and facilitates the adsorption of dye molecules, thereby promoting photocatalytic reactions [23,57].

The comparison also indicates that the incorporation of dopant cations with a lower valence than Zr^4+^ generally enhances the photocatalytic activity of ZrO_2_. Regardless of the dopant species, the improvement is commonly attributed to the generation of lattice defects, particularly oxygen vacancies, which promote charge-carrier separation and reduce electron–hole recombination. In addition, dopants may stabilize metastable crystalline phases, decrease crystallite size, and modify the electronic structure, thereby increasing the availability of reactive oxygen species involved in the degradation process [18,38,41].

In contrast, ZrO_2_ thin films usually exhibit lower degradation efficiencies, typically ranging from approximately 16% to 46% under comparable UV irradiation conditions. This behaviour is primarily associated with the lower exposed surface area and the reduced number of accessible active sites compared with nanopowders. Nevertheless, thin-film photocatalysts offer several important practical advantages, including excellent adhesion to the substrate, superior mechanical and chemical stability, easy recovery after photocatalytic treatment, and the possibility of repeated use without requiring additional separation or filtration steps [18,38,58].

The Y-doped ZrO_2_ thin films developed in the present work exhibit photocatalytic efficiencies that are among the highest reported for sputtered zirconia coatings under UV irradiation, despite the inherently lower specific surface area of thin films compared with nanoparticle photocatalysts. For RhB degradation, the Y3 sample achieves nearly complete discoloration within 50 min, whereas both Y0 and Y5 exhibit lower efficiencies, demonstrating that an optimum yttrium concentration exists for maximizing photocatalytic activity.

## 3. Materials and Methods

Using the radio frequency magnetron sputtering (RFms, home-made, Iasi, Romania) method (power 100 W), yttrium-doped ZrO_2_ thin films with increasing concentrations were deposited on unheated ITO/glass substrates (Merck KGaA, Darmstadt, Germany). The procedure is described as follows: A high-purity (99.95%) zirconium target (Sigma-Aldrich, St. Louis, MO, USA) (5 cm diameter and 0.3 cm thickness) was positioned 7 cm from the substrate. The chamber achieved a base pressure below 10^−8^ Pa using an Agilent turbomolecular pump backed by a dry scroll pump. Ar was used as the sputtering gas (50 sccm) and O_2_ (1 sccm) as the reactive gas, the total pressure being kept at 0.8 Pa. Sets of 1, 3 and 5 Y_2_O_3_ (Sigma-Aldrich, St. Louis, MO, USA) discs (2.5 mm diameter) were placed on the Zr target to obtain Y-doped ZrO_2_ films with increasing Y content. The deposition time was 60 min. The as-deposited thin films were subjected to heat treatment to compact the structure and increase the degree of crystallinity. They were heat-treated in air for 4 h at 400 °C using a temperature-controlled furnace Microterm 1206 (Caloris Group, Bucharest, Romania), the heating rate being ≈ 100 °C/h. The cooling was performed with the furnace naturally.

Using a Shimadzu 6000 diffractometer in θ-2θ geometry (40 kV, 30 mA, CuK_α_ radiation (1.540591 Å)) (Shimadzu Scientific Instruments Inc., Columbia, MD, USA), the structures of the heat-treated films and those of the heat-treated ITO substrate were analyzed. The Scherrer formula was applied to calculate the average crystallite sizes [60].

Surface analysis was conducted via X-ray photoelectron spectroscopy (XPS) using a Physical Electronics-Ulvac (PHI 5000 VersaProbe) spectrometer (Ulvac-PHI Inv., Chikasaki, Japan), equipped with a monochromated Al Kα source (1486.6 eV, 25 W). This characterization provided detailed insights into the elemental composition and the chemical states of the atomic species at the film surface.

The surface morphology of the thin films was investigated using a Solver PRO scanning probe microscope from NT-MDT, Moscow, Russia operating in non-contact mode.

The thickness of the films was determined with a Dektak XT stylus profilometer (Bruker, Wissembourg, France) and found to be 100 nm.

The optical bandgap (E_g_), an important parameter defining the light absorption range of the material, was determined for all the films using diffuse reflectance spectroscopy (DRS). Spectra were recorded in the 190–1100 nm range with a Camspec 501M spectrophotometer featuring a BaSO_4_-coated integrating sphere (Spectronic CamSpec Ltd., Leeds, UK) the ITO substrate served as the reference (blank). Based on the Kubelka–Munk theory and the characteristic direct allowed transition of ZrO_2_, the bandgap energies were estimated by extrapolating the linear portion of the (Fhν)^2^ vs. hν plots to the energy axis intercept (F is the Kubelka–Munk function) [61].

Wettability and photo-induced hydrophilicity were characterized using a Data Physics OCA 15EC system (DataPhysics Instruments GmbH, Filderstadt, Germany) equipped with a 150 W mercury lamp for UV activation. The study involved tracking the evolution of deionized water contact angles through a photoactivation stage until reaching the steady-state saturation, followed by a dark deactivation stage. Detailed measurement protocols are available in reference [61].

Photocatalytic degradation of RhB (97%, Sigma-Aldrich, St. Louis, MO, USA), and MB (98%, Sigma-Aldrich, St. Louis, MO, USA)was investigated for concentrations of 1, 3, and 5 mg/L using a Camspec 501M spectrophotometer. All solutions were weakly acidic, with pH = 5.8–5.5 for RhB and pH = 6.6–6.5 for MB. The removal efficiency was derived from the decrease in the characteristic absorption peak at 553 nm for RhB and at 663 nm for MB.

To ensure adsorption–desorption equilibrium, the photocatalysts were immersed in 10 mL of RhB or MB solution and kept in the dark for 1 h before light exposure. Under irradiation with a 150 W mercury lamp, without any additional optical filters, the degradation process was monitored every 20 min for RhB and every 5 min for MB. The distance between the light source and the photocatalyst films was maintained at 10 cm. The experiment was continued until the difference in absorbance values was less than 1%.

The kinetic aspects of RhB and MB photodegradation over undoped and Y-doped ZrO_2_ films were studied to clarify the basic reaction mechanism. It was confirmed for all the films that the degradation followed a pseudo-first-order model, showing the highest R^2^ correlation coefficient. Consequently, the apparent rate constant (k_1_) and the degradation efficiency (DE) were derived as follows:

(16) $$ln\frac{C_{t}}{C_{0}}=-k_{1}\cdot t,$$

and

(17) $$DE\left(\%\right)=\frac{C_{0}-C_{f}}{C_{0}}100.$$

In these expressions, C_0_ and C_f_ correspond to the dye concentrations before and after the photocatalytic process, while C_t_ signifies the instantaneous concentration at the moment in time, t.

The operational stability of the films was tested over four successive photocatalytic cycles. Following each cycle, the samples were cleaned with ethanol and water and dried at 70 °C for 2 h. The photocatalytic performance was recorded after each step to determine the degradation of the films’ activity over time.

## 4. Conclusions

In this study, the hydrophilic and photocatalytic activities of undoped and yttrium-doped ZrO_2_ thin films were successfully evaluated. The films, deposited via RF magnetron sputtering onto ITO/glass substrates, demonstrated significant structural changes upon doping.

The XRD results confirm the presence of the monoclinic phase in the undoped ZrO_2_ film, with a crystallite size of 6.55 nm, which decreased to 6.01 nm as the dopant was introduced, the observed differences being advantageous for the investigated photocatalytic performances. We achieved the desired stabilization of the tetragonal ZrO_2_ phase in the yttrium-doped films, while the orthorhombic phase was also evidenced in the sample with a lower yttrium content.

This structural transition, along with a higher concentration of oxygen vacancies and lattice defects induced by these phases, is identified as the key factor behind the superior photocatalytic performance observed in the doped samples across all tested concentrations of RhB and MB dyes.

By doping with yttrium, the optical bandgap of ZrO_2_ slightly increases, confirming existing literature data. Consequently, from this perspective, the desired photocatalytic property—a more efficient utilization of the visible light spectrum—is not achieved. The relative positions of the edges of the valence and conduction bands for the films favour the water oxidation process, so the holes play an important role in the photocatalytic process. The ITO substrate does not contribute to the separation of photogenerated charge carriers.

By yttrium doping, in contrast to the undoped films, these become superhydrophilic. Superhydrophilicity is achieved after 200 min of irradiation, reaching contact angles of 9 deg.

The photodegradation time of RhB and MB increases proportionally with the dye solution concentration, while the degradation efficiency values decrease with the solution concentration for all three studied layers. The photocatalytic activity of the yttrium-doped thin films is superior to that of the undoped layer, likely due to the presence of the tetragonal phase, reaching degradation efficiencies of 100% after 40 min of irradiation (for 1 mg/L RhB) for both doped samples, while in the case of 1 mg/L MB degradation, values of over 93% are achieved in just 20 min of irradiation. The sample with lower yttrium content (0.2Y3) shows enhanced degradation efficiency for both RhB and MB, and is also the most stable, which is attributed to more oxygen vacancies and defects on the film surface. The photodegradation mechanism is driven by photogenerated holes.

## Figures and Tables

**Table 1:** Average crystallite sizes (D) for both the studied films and their ITO substrates.

Sample	Crystalline Structure	D (nm)
ITO substrate [5]	ITO	34.33
Y0	m-ZrO_2_	6.55
	ITO	34.35
Y3	m-ZrO_2_	6.21
	o-ZrO_2_	36.25
	ITO/t-ZrO_2_	30.11
Y5	m-ZrO_2_	6.01
	ITO/t-ZrO_2_	24.58

**Table 2:** Chemical composition, valence band energy and conduction band energy.

Sample	Molar Formula	*E_VB_* (eV)	*E_CB_* (eV)
Y0	ZrO_2_	2.988	−0.158
Y3	Zr_0.9911_Y_0.0089_O_1.9955_	3.011	−0.191
Y5	Zr_0.9617_Y_0.0383_O_1.9809_	3.057	−0.266

**Table 3:** Comparative analysis of the photocatalytic performance of ZrO_2_-based photocatalysts reported in the literature.

Material	Pollutant	Light Source	Time (min)	Degradation	k_app_ (min^−1^)	Reference
ZrO_2_ thin films	MB	UV	100	16%	0.109	[18]
MnPOM/ZrO_2_ thin films				43%	0.151	
0YSZ nanoparticles	RhB	UV	40	70.4%	0.024776	[20]
Y doped t-ZrO_2_ nanoparticles			40	~98%	0.035190	
YSZ (Zr_0.72_Y_0.28_O_1.86_) Nanopowder (400 mg)	MB	UV	180	90%	-	[23]
0YSZ	Acid Orange 7	UV	90	84%	0.0204	[24]
8YSZ (30 mg)			90	99.1%	0.05	
ZrO_2_ thin films	MB	UV	100	16%	0.0015	[38]
CrPOM/ZrO_2_ thin films				46%	0.0057	
ZrO_2_	MB	Visible	1440	68%	0.00173	[41]
Nd(9%)–ZrO_2_ nanoparticles			120	99%	0.00733	
t-ZrO_2_ nanoparticles	MB	Solar	300	89.1%	0.007	[57]
ZrO_2_ thin films, 550 °C	MB	UV	600	36%	0.0005	[58]
Nd(2%)–ZrO_2_ nanoparticles	MB	Visible	120	90%	0.0144	[59]
	RhB	Visible	120	70%	0.00944	
Y-doped ZrO_2_ thin films (200 mm^2^)	RhB (3mg/L)	UV	50	Y0-83.17%	0.03369	This work
				Y3-90.14%	0.05009	
				Y5-84.47%	0.04056	
	MB (3 mg/L)	UV	50	Y0-95.02%	0.0499	
				Y3-99.7%	0.09843	
				Y5-99.7%	0.08679	

## Data Availability

The original contributions presented in this study are included in the article. Further inquiries can be directed to the corresponding authors.
